# Sustainable Hybrid Laminated Composites Reinforced with Bamboo, Flex Banner, and Glass Fibers: Impact of CaCO_3_ Filler on Mechanical Properties

**DOI:** 10.3390/polym18020275

**Published:** 2026-01-20

**Authors:** Rahmat Doni Widodo, Muhammad Irfan Nuryanta, Prima Astuti Handayani, Rizky Ichwan, Edi Syams Zainudin, Muhammad Akhsin Muflikhun

**Affiliations:** 1 Department of Mechanical Engineering, Universitas Negeri Semarang, Semarang 50229, Indonesia; irfannuryanta@mail.unnes.ac.id; 2 Department of Chemical Engineering, Faculty of Engineering, Universitas Negeri Semarang, Semarang 50229, Indonesia; prima@mail.unnes.ac.id (P.A.H.); rizkyichwan21@students.unnes.ac.id (R.I.); 3 Advanced Engineering Materials and Composite Research Centre (AEMC), Department of Mechanical and Manufacturing Engineering, Universiti Putra Malaysia (UPM), Serdang 43400, Selangor, Malaysia; edisyam@upm.edu.my; 4 Department of Mechanical and Industrial Engineering, Faculty of Engineering, Universitas Gadjah Mada, Jl. Grafika No. 2, Yogyakarta 55281, Indonesia; akhsin.muflikhun@ugm.ac.id

**Keywords:** hybrid composites, resource efficiency, CaCO_3_ filler, natural fiber, SDG 12

## Abstract

The increasing demand for sustainable polymer composites has driven the development of hybrid laminates that combine natural, recycled, and synthetic reinforcements while maintaining adequate mechanical performance. However, the combined influence of stacking sequence and mineral filler addition on the mechanical behavior of such sustainable hybrid systems remains insufficiently understood. In this study, sustainable hybrid laminated composites based on epoxy reinforced with glass fiber (G), bamboo fiber (B), and flex banner (F) were fabricated with varying stacking sequences and calcium carbonate (CaCO_3_) filler contents (0 and 1 wt.%). A total of nine laminate configurations were produced and evaluated through flexural and impact testing. The results demonstrate that mechanical performance is strongly governed by laminate architecture and filler addition. The bamboo-dominant G/B/B/B/G laminate containing 1 wt.% CaCO_3_ exhibited the highest flexural strength (191 MPa) and impact resistance (0.766 J/mm^2^), indicating a synergistic effect between reinforcement arrangement and CaCO_3_-induced matrix strengthening. In contrast, the lowest performance was observed for the G/F/B/F/G configuration without filler. Overall, all hybrid composites outperformed neat epoxy, highlighting the potential of bamboo–flex banner hybrid laminates with CaCO_3_ filler for sustainable composite applications requiring balanced mechanical properties. This work aligns with SDG 12 by promoting resource-efficient circular-economy practices through the utilization of flex banner material and natural fibers as reinforcements in epoxy-based hybrid composites.

## 1. Introduction

Polymer matrix composites have been widely developed over the last several decades in response to the demand for lightweight, high-mechanical-property materials that are more environmentally friendly in various industries, including transportation, construction, and automotive engineering [1,2,3,4,5]. These materials can be modified to demonstrate higher stiffness, strength, and durability than monolithic polymers by adding one or more reinforcing phases to a polymer matrix [1,6]. Simultaneously, the growing pressure on the environmental impact of engineering materials, exerted by both the government and society, has triggered the advent of renewable fibers, recycled polymer-based substances, and mineral fillers as a more sustainable alternative to fully synthetic systems [2,3,4].

Natural lignocellulosic fibers have garnered growing interest as a polymer composite reinforcement material due to their low density, high specific strength and stiffness, abundance, and biodegradability [5,7]. Particularly, bamboo fibers have potential as a fast-growing plant; bamboo is more widely available and can deliver good mechanical performance at a low cost. In woven structures, three-dimensional networks of bamboo fibers can be created that ensure effective load transfer, in-plane stiffening, and impact resistance in laminate composites [5,7]. Simultaneously, numerous flexible banners made of plastic are produced as solid waste products of the outdoor promotional media. These signs are typically made from thermoplastic, waterproof sheets and are not recyclable, leading to their accumulation in landfills [8]. Their inclusion as interlayers in polymer composites provides a possible avenue for utilizing this waste material, while also providing tensile and flexural reinforcement and enhancing the compactness of the laminate structure [8]. Conversely, fiberglass has remained one of the most popular synthetic reinforcements in the composite industry due to its high tensile strength, good stiffness, and stability under various service conditions [9,10]. As a top layer in hybrid laminates, fiberglass has the capability of increasing surface strength and stiffness while protecting the inner layers made from more sustainable materials.

The hybridization of various reinforcements in a single laminated composite enables the synergistic integration of individual benefits. Specifically, hybrid laminates comprising natural fibers, recycled polymer sheets, and synthetic fibers present a high potential for achieving an acceptable combination of mechanical performance, durability, and environmental footprint [11,12,13]. The general behavior of these hybrid systems is highly dependent on the order and arrangement of the reinforcing layers, which control stress redistribution, damage creation, and failure mode during mechanical loading [14,15,16,17,18]. Besides the reinforcement architecture, the addition of inorganic fillers, such as calcium carbonate (CaCO_3_), to the polymeric structure also enables the control of the composites’ characteristics. CaCO_3_ has been a popular, low-cost, and non-toxic filler that contributes to stiffness, alters rheological properties during processing, and reduces overall material cost [19,20]. Small CaCO_3_ particles may partially fill the microscopic pores in the matrix, thereby enhancing the interfacial contact between matrix fibers, reducing moisture ingress routes, and affecting crack propagation [21]. Nevertheless, the performance of CaCO_3_ in composite laminates is highly dependent on particle size, dispersion, concentration, and the interaction between the particles and the various reinforcing layers [19,22,23]. Limited systematic studies exist on the evaluation of these effects in combination in sustainable hybrid laminates.

Table 1 summarizes related studies and shows how this work differs from previous research. Earlier studies have examined woven bamboo laminates [5,7], recycled flex-banner interlayers [8], and fiberglass-based hybrid laminates [9,10]. Other studies also show that laminate performance depends strongly on the stacking sequence [10,14,15,16,17,18]. In addition, CaCO_3_-filled epoxy systems can affect interfacial behavior, but the outcome depends on filler dispersion and content [19,20,21,22,23]. However, studies that combine bamboo, recycled flex banner, and fiberglass in one laminate system and evaluate the joint effects of stacking sequence and low CaCO_3_ content across multiple properties are still limited.

Therefore, the present work investigates laminated hybrid polymer composites reinforced with three categories of materials: woven bamboo, recycled flex banner, and fiberglass. The article examines the sequence of these reinforcing layers and the addition of CaCO_3_ filler into the polymer matrix, focusing on the density, flexural strength, impact resistance, and hardness of the resulting composites. Laminated hybrid composites reinforced with bamboo, recycled flex banner, and fiberglass were fabricated with different layer configurations. The polymer matrix was prepared with 1 wt.% CaCO_3_. The effects of layer sequence and filler addition on the physical and mechanical properties were then evaluated. The primary objective is to develop sustainable hybrid composite structures incorporating natural and recycled reinforcements in combination with traditional synthetic fibers, which produce competitive performance suitable for use in construction, automotive, and other engineering sectors, thereby reducing waste and enhancing resource efficiency.

## 2. Materials and Methods

### 2.1. Materials

The main materials used in this work were glass fiber, bamboo matting, and a flex banner. The flex banner reinforcement used in this study was sourced from local advertising printing service providers in Semarang, Indonesia, and had a measured density of 1.629 g/cm^3^. The flex banner was cut into two parts, with 230 mm × 180 mm and 200 mm × 7 mm. The bamboo reinforcement consisted of bamboo apus (*Gigantochloa apus*), which was sourced, processed, and woven by local craftsmen in Semarang. Woven glass fiber with an areal weight of 600 g/m^2^ (WR 600), epoxy resin, and the corresponding hardener were supplied by Justus Kimia chemical store, Semarang, Indonesia. Calcium carbonate (CaCO_3_) filler was obtained from Karya Agung chemical supplier, Semarang, Indonesia.

**Figure 1:**
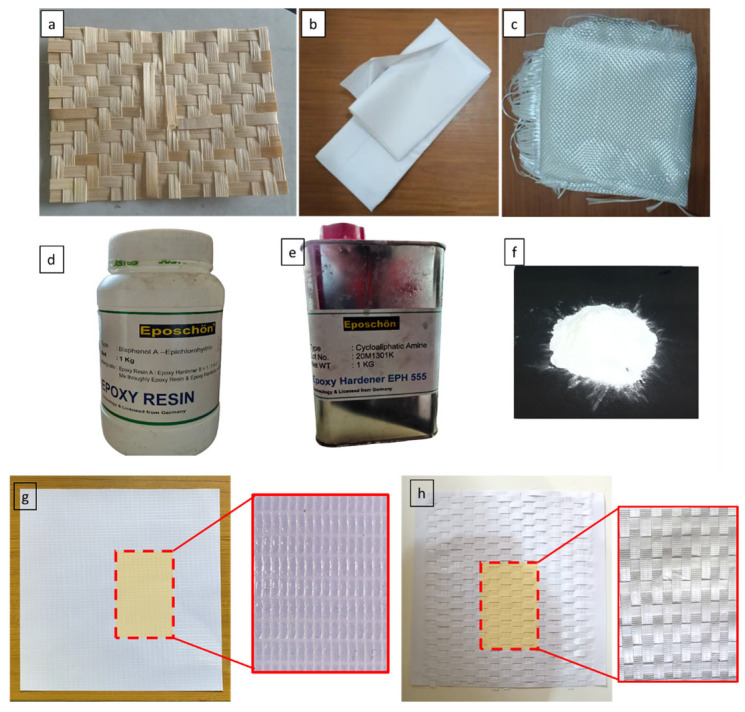
Materials used in this study: (**a**) Bamboo, (**b**) Flex banner, (**c**) Glass Fiber, (**d**) Epoxy, (**e**) Hardener, (**f**) CaCO_3_ filler, (**g**) Flex banner before weaving, (**h**) Flex banner after weaving.

First, the piece was cut into multiple narrow strips, and a spacing of approximately 7 mm was provided between the cuts. Then, the second piece was woven with the first to create a 20 mm × 15 cm weave, as shown in Figure 1g,h. The matrix material used was a bisphenol-A-type epoxy resin mixed with EPH 555 hardener at a ratio of 3:1. Additionally, CaCO_3_ filler is utilized to enhance the physical and mechanical properties of the composite [24]. Additional materials, such as sealant tape, paper tape, peel ply, flow media, vacuum plastic, spirals, and hoses, were also used to support the composite manufacturing process [11]. Figure 1 shows the materials used in this study.

### 2.2. Method

#### 2.2.1. Bamboo Alkalization

**Figure 2:**
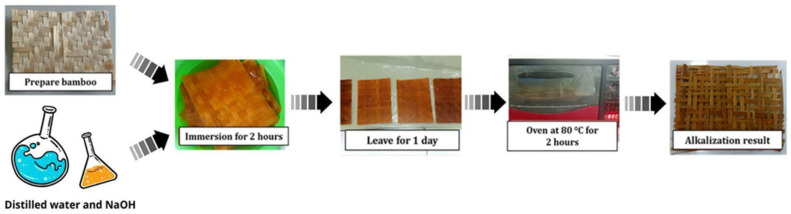
Bamboo alkalization process.

Figure 2 illustrates the sequence of alkali treatment (alkalinization) applied to bamboo woven fabric using a NaOH solution to improve the fiber surface prior to its use as composite reinforcement. First, the bamboo weave was cut to the required size and cleaned to remove dust and contaminants. Distilled water was prepared as the solvent, and NaOH pellets were weighed to obtain a 5 wt.% solution, then gradually dissolved in distilled water under continuous stirring to form an alkaline solution. Because the dissolution is exothermic, appropriate precautions were taken during preparation. The bamboo weave was then fully immersed in the NaOH solution for 2 h to partially remove hemicellulose, lignin, and residual surface impurities, while promoting a rougher and cleaner fiber surface [25].

After immersion, the bamboo was rinsed with distilled water until the rinse water approached neutral pH and then conditioned at room temperature for more than 24 h to allow excess moisture to evaporate. The samples were subsequently oven-dried at 80 °C for 2 h under adequate air circulation to minimize the formation of microbubbles. After treatment, the bamboo weave exhibited a darker color, indicating the removal of non-cellulosic components and modification of the fiber surface [26]. This treatment is expected to improve fiber–matrix adhesion during composite fabrication by providing a cleaner and rougher surface that promotes mechanical interlocking and interfacial bonding [27].

#### 2.2.2. Composite Manufacturing

**Figure 3:**
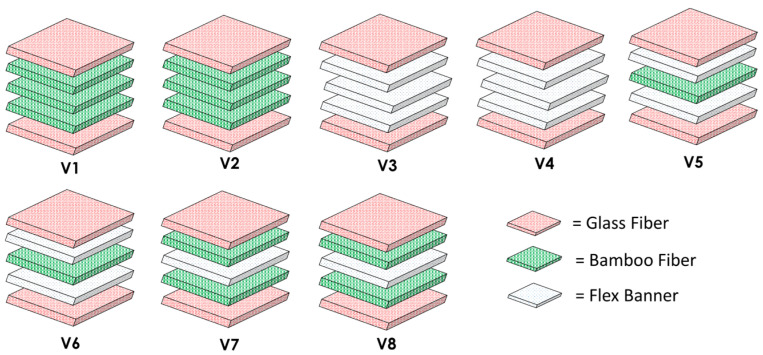
Schematic illustration of stacking sequences for all laminate configurations with and without CaCO_3_ filler.

Composite laminates were fabricated using the VARI technique [28]. First, the mold surface was cleaned and coated with a suitable release agent. The reinforcing layers were then arranged on the mold according to the desired stacking sequences (Figure 3), using combinations of glass fiber (G), bamboo (B), and woven flex banner (F). The different laminate configurations and CaCO_3_ contents (0 and 1 wt%) are summarized in Table 2, where V1–V8 denote the hybrid laminates, and V9 corresponds to the neat epoxy laminate.

**Figure 4:**
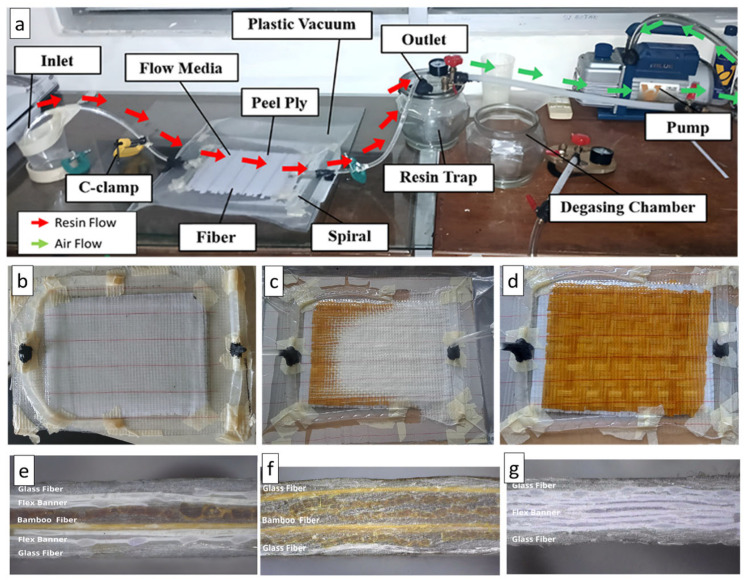
Photographic documentation of the vacuum-assisted resin infusion (VARI) process and results: (**a**) experimental setup, (**b**) initial stage, (**c**) infusion stage, (**d**) complete infusion stage, (**e**) composite V1, (**f**) composite V5, (**g**) composite V4.

After stacking the dry reinforcements, peel ply and flow media were placed on top of the lay-up, followed by installation of spiral tubing at the resin inlet and outlet. The assembly was sealed with vacuum bagging film and sealant tape, and the vacuum lines were connected to a vacuum pump [29,30,31]. The system was evacuated until a stable vacuum was achieved, ensuring that no air leakage was present. At this stage, the fibers remained dry, as indicated by their uniform white appearance (Figure 4b).

The epoxy resin and hardener were mixed thoroughly, and the predetermined amount of CaCO_3_ filler (0 or 1 wt%) was added to the resin and hardener mixture. The dispersion of CaCO_3_ filler in the resin matrix was carried out using a mechanical mixer. The resin and CaCO_3_ were mixed at a constant speed of 250 rpm for 10 min at room temperature to obtain a homogeneous mixture [24]. The filled resin was then infused into the evacuated lay-up through the inlet spiral under vacuum pressure. During infusion, the resin front progressed across the laminate, wetting the reinforcements and changing their color from white to a darker shade (Figure 4c). The infusion was continued until all regions of the laminate were fully impregnated, as evidenced by a uniform appearance across the entire surface (Figure 4d). The laminate was maintained under vacuum and allowed to cure at room temperature for 24 h [11].

**Figure 5:**
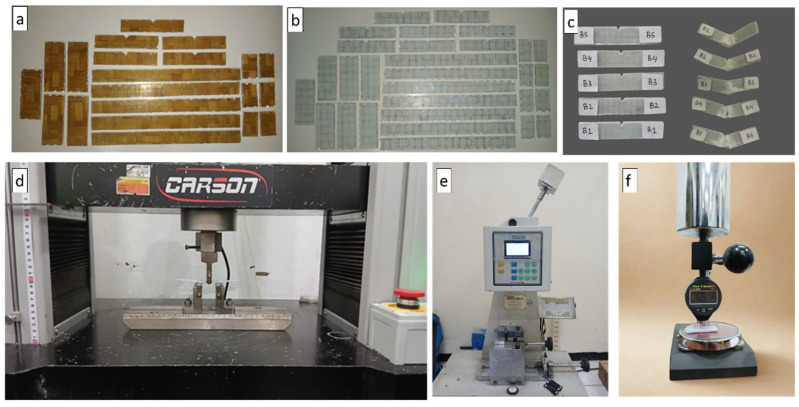
(**a**) V1 testing specimen (**b**) V4 testing specimen (**c**) Before and after impact testing (**d**) Universal testing machine (**e**) Impact testing (**f**) Hardness testing.

After curing, the composite panel was removed from the mold, and excess edges were trimmed. Specimens for mechanical and physical testing were cut from the panels using a diamond saw in accordance with the relevant ASTM standards. The specimen dimensions were 25 mm × 15 mm × 3 mm for density measurements, 64 mm × 12.7 mm × 3.2 mm for the Izod impact test (ASTM D256 [32]), 150 mm × 12.7 mm × 3.5 mm for the three-point bending test (ASTM D790 [33]), and 50 mm × 20 mm × 10 mm for hardness testing using the Shore D method (ASTM D2240 [34]). For each laminate configuration and test condition, five specimens were tested to ensure statistical reliability. The reported results represent the mean values of the measured data, accompanied by the corresponding standard deviations. Data variability is illustrated using error bars in the graphical presentations. The testing equipment used for flexural, impact, and hardness measurements is shown in Figure 5d–f.

## 3. Results and Discussion

### 3.1. Composite Density

**Figure 6:**
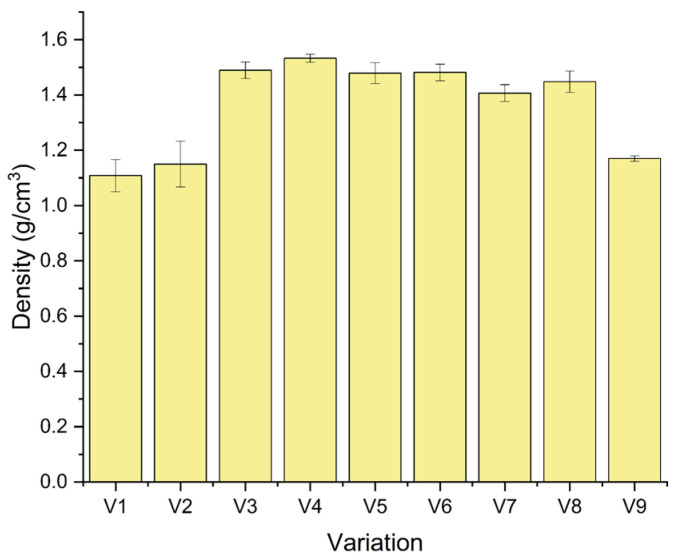
Density value of the composite.

Figure 6 shows the density of the laminates listed in Table 2. The measured values range from 1.11 to 1.53 g/cm^3^. The neat epoxy reference (V9) has a density of 1.17 g/cm^3^, which is consistent with typical values for bisphenol-A epoxy systems. Bamboo-dominant laminates V1 and V2, with G/B/B/B/G and G/B/B/B/G stacking sequences, have densities of 1.11 and 1.15 g/cm^3^, corresponding to reductions of 5.2% and 1.7% compared with neat epoxy, respectively. This pattern indicates the low specific gravity of bamboo compared to other reinforcements. In V2, the addition of 1 wt.% CaCO_3_ increases the density by approximately 0.04 g/cm^3^ (3.7%), which may be attributed to the higher density of CaCO_3_ and a reduction in microvoid content through particle-assisted packing within the matrix [35].

Laminates dominated by flex banner and glass fiber (V3–V6) exhibited substantially higher densities. For the G/F/F/F/G sequence, the density increased from 1.49 g/cm^3^ in V3 to 1.53 g/cm^3^ in V4 when 1 wt% CaCO_3_ is added, which corresponds to an increase of 0.04 g/cm^3^ (2.9%) [36]. These values were about 27–31% higher than those of neat epoxy, reflecting the intrinsically higher densities of fiberglass, flex banner, and CaCO_3_ compared with the polymer matrix [37,38]. For the mixed hybrid sequences G/F/B/F/G (V5, V6) and G/B/F/B/G (V7, V8), the densities ranged from approximately 1.41 to 1.48 g/cm^3^. The incorporation of 1 wt.% CaCO_3_ resulted in a marginal increase in density, with changes of about 0.2% for the G/F/B/F/G sequence and approximately 3.0% for the G/B/F/B/G sequence (Table 3).

Overall, the results demonstrate that composite density is primarily governed by the proportion of high-density reinforcements (glass fiber, flex banner, and CaCO_3_) in the stacking sequence, with bamboo-rich laminates yielding lower densities and flex-rich laminates yielding the highest values. A slight and consistent increase in density was observed after CaCO_3_ addition. The trend suggests improved matrix densification and packing. This densification may contribute to changes in mechanical response and dimensional stability of the hybrid laminates under the present processing and testing conditions [39].

### 3.2. Volume Fraction Analysis

**Figure 7:**
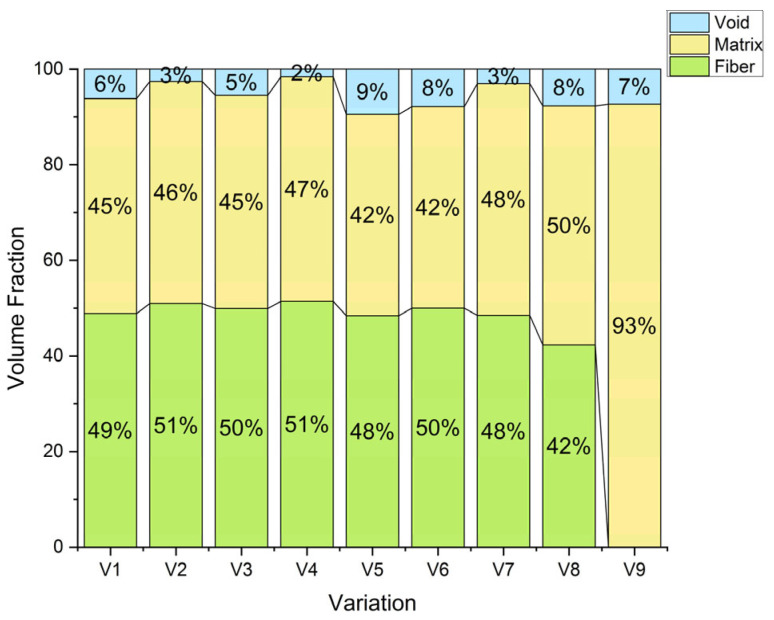
Volume fraction of the composite.

Figure 7 presents the fiber, matrix, and void volume fractions of the hybrid laminates with different stacking sequences and CaCO_3_ contents. The measured fiber fractions lie between about 42 and 51%, the matrix fractions between 45 and 51%, and the void fractions between 2 and 8%. These results confirm that both stacking arrangement and CaCO_3_ addition strongly influence the internal structure and compactness of the composites [40].

In the case of the bamboo-dominant laminates V1 and V2 (G/B/B/B/G), the fiber content was approximately 49% and 51%, respectively, while the matrix content were 45% and 46%, and the void fractions was 6% and 3%, respectively. Therefore, the addition of 1 wt% CaCO_3_ in V2 increased the fiber fraction by approximately 2 vol% and reduce the void fraction by approximately 3 vol% over V1, but does not cause a significant change in the matrix fraction. This action implies that the CaCO_3_ filler may have improved the resin flow and wettability of the bamboo fibers, resulting in improved packing and a reduction in the number of microvoids in the laminate [11]. This effect may be explained by the fact that fine CaCO_3_ particles can partially fill the interstitial space of the matrix, thereby enhancing packing efficiency during curing [39].

The same tendency was observed in the flex-banner/fiberglass-dominated laminates in larger proportions. The fiber, matrix, and void fractions in V3 (G/F/F/F/G without filler) were approximately 50%, 45%, and 5%, respectively. In V4 (with 1 wt% CaCO_3_), these values were approximately 51%, 47%, and 2%, respectively. The fact that the void content was decreased by over half, along with the increase in the fiber and matrix fractions, is clear evidence of better consolidation of the laminate. The latter findings are in line with the increased density observed for V4 and suggests improved impregnation and consolidation. Resin penetration and interfacial bonding are may be facilitated by the smooth and uniform surfaces of the flex banner and fiberglass, which are easier to impregnate than the more irregular bamboo fibers [41,42].

For the mixed hybrid configurations that alternate bamboo, flex banner, and fiberglass (V5–V8), the fiber fractions were in the range of 48–51% for V5 and V6, but decreased to approximately 48% and 42% for V7 and V8, respectively. At the same time, the void fraction increased from about 2–3% in V5 and V6 to 8% in V7 and 7–8% in V8. The lower fiber content and higher void content in V7 and V8 indicate less uniform bonding between the different reinforcement types. This heterogeneity is likely caused by the other surface energies, morphologies, and absorbability of natural (bamboo) and synthetic (fiberglass and flex banner) fibers, which can hinder resin flow and reduce interfacial adhesion. As expected, the neat epoxy control V9 contains no fibers and consists of 93% matrix and 7% voids.

Overall, the addition of 1 wt% CaCO_3_ systematically reduces the void fraction and slightly increases the adequate fiber and matrix fractions in almost all stacking sequences. Among all laminates, V4 exhibits the best consolidation, characterized by the highest fiber fraction (51%) and the lowest void fraction (2%) [43]. A dense microstructure is generally associated with improved mechanical performance, particularly flexural strength. Voids are known to act as stress concentrators and crack initiation sites under load. Therefore, the appropriate optimization of the stacking sequence, combined with a modest amount of CaCO_3_ filler, is a crucial step in designing dense and mechanically robust hybrid composites [44].

### 3.3. Impact Strength Analysis

**Figure 8:**
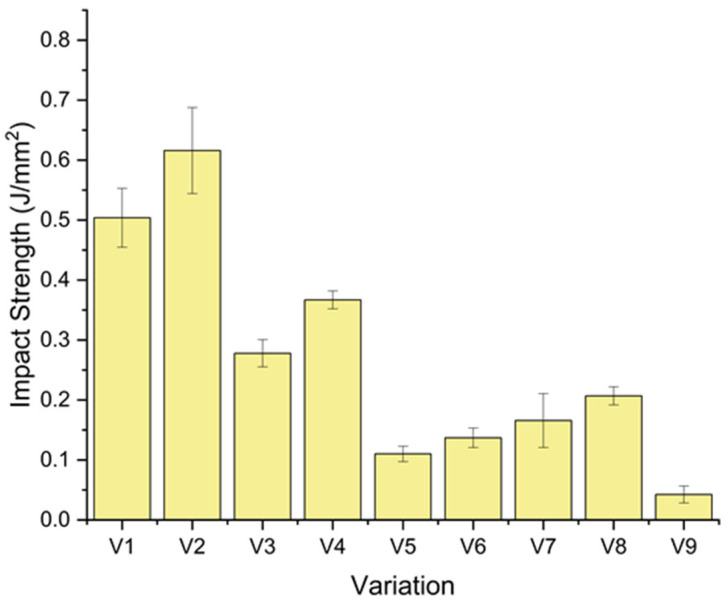
Impact strength of the composite.

Figure 8 shows the impact strength of the laminates as a function of stacking sequence and CaCO_3_ addition. The recorded values range from about 0.04 to 0.62 J/mm^2^. The fiber-reinforced composites exhibited higher impact strength compared to the neat epoxy (V9), which has an impact strength of only 0.042 J/mm^2^. Depending on the layer arrangement, the reinforced laminates exhibited impact strengths up to 14.5 times higher than the neat epoxy matrix. This trend suggests that fiber reinforcement and, where applicable, CaCO_3_ addition increased the energy-absorption capability of the epoxy-based system under impact loading.

The highest impact strength was obtained for the bamboo-dominant laminate V2 (G/B/B/B/G + 1 wt% CaCO_3_), with a value of about 0.616 ± 0.072 J/mm^2^, followed by its unfilled counterpart V1 (0.504 ± 0.049 J/mm^2^). The introduction of 1 wt% CaCO_3_, therefore, increased the impact strength of the bamboo laminate by 22% over that of V1. This behavior may be attributed to the relatively ductile response of bamboo fibers, which enables the dissipation of energy during crack propagation through fiber pull-out, deformation of the microfibrils, and interfacial friction mechanisms [45]. This CaCO_3_ filler may also improve interfacial bonding between bamboo fibers and the epoxy matrix, resulting in decreased strength and improved stress transfer, which leads to greater energy absorption at impact loading [46].

The flex banner and fiberglass (V3 and V4, G/F/F/F/G) dominated laminates exhibited intermediate impact strengths of 0.278 ± 0.023 and 0.367 ± 0.015 J/mm^2^, respectively. These values were 6.5–8.6 times higher than that of neat epoxy, but lower than that of the bamboo-rich laminates. Fiberglass and flex banner are comparatively rigid and less compliant materials, rendering them more brittle and limiting their capacity to absorb impact energy [47,48]. However, addition of 1 wt% CaCO_3_ in V4 raises the impact strength by about 32 percent of V3, which implies that the filler improves the compaction of the matrix and quality of the interface, even in synthetic-fiber-dominant compositions. The trade-off between rigidity and energy absorption in composite materials having a high content of synthetic fibers is common to this behavior [49].

The reinforced laminates with the lowest impact strengths were the hybrid stacking sequences that include bamboo, flex banner, and fiberglass (V5–V8), with the range of values between 0.11 and 0.21 J/mm^2^. The growth rate brought about by CaCO_3_ is also noticeable: V6 and V8, which are impregnated with 1 wt% CaCO_3_, have about a 25% jump in impact strength compared to V5 and V7, which are not impregnated with the filler. Nevertheless, with or without filler, these laminates are not as tough as the bamboo-dominant or flex-dominant system. It is possible to explain the reduced impact performance by the incompatible mechanical properties and the non-uniform interfaces of the various reinforcements. The dissimilarity in surface energy, surface morphology, and wettability between natural bamboo fibers and synthetic fiber glass/flex banner layers prevents homogeneous resin flow and interfacial adhesion. It encourages stress concentrations, premature crack formation, and interlaminar delamination under impact loading [50,51].

The results of impact show that the sequence of stacking as well as the addition of CaCO_3_, affected on the energy absorption capacity of the hybrid laminates. Bamboo-dominated arrangements tended to be more effective in absorbing impact energy, while mixed hybrid lay-ups tend to experience interfacial incompatibilities. The steady increase in impact strength (by about 22–32%) when 1 wt.% CaCO_3_ is added to all types of laminated composites testifies the positive contribution of the filler to reinforcing the strength of fiber–matrix interfaces and increasing the impact resistance of the hybrid polymer composites.

### 3.4. Flexural Strength Analysis

**Figure 9:**
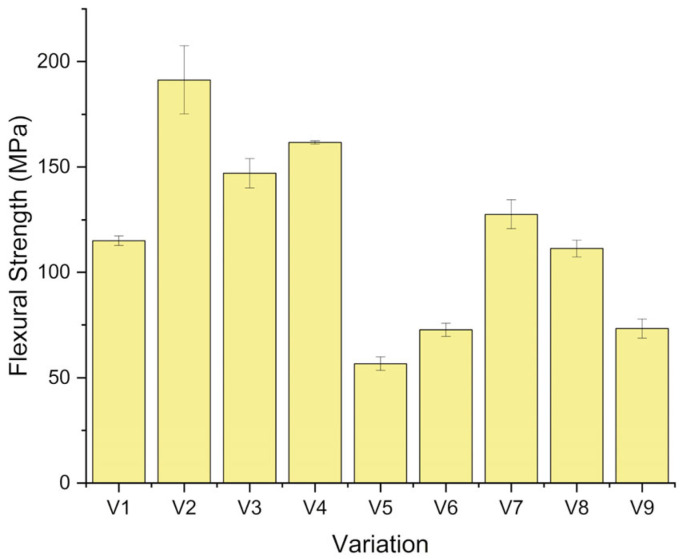
Flexural strength of the composite.

The flexural test outcomes (Figure 9) indicate that the bending strength of the laminates was significantly influenced by the type of reinforcement structure and the presence of CaCO_3_ filler [52,53]. The flexural strengths measured ranged from 61 to 191 MPa. All the reinforced laminates except V5 exhibited higher flexural strength than the neat epoxy (V9, 73 ± 5 MPa). This demonstrates the positive effect of fiber reinforcement on load-carrying ability during bending.

The maximum flexural strength was observed for the bamboo-dominated laminate V2 (G/B/B/B/G, 1 wt% CaCO_3_), with a value of 191 ± 16 MPa. Its unfilled counterpart (V1) reaches 115 ± 2 MPa. Therefore, as 1 wt% CaCO_3_ is added, the flexural strength of the bamboo laminate is boosted by about 76 percent. This significant difference indicates that CaCO_3_ has a substantial may have enhanced the interfacial strength between bamboo fibers and the epoxy matrix. Microvoids at the fiber–matrix interface can be partially filled by well-dispersed filler particles, which can eliminate local stress concentrations and enhance the effective transfer of stress during bending. Additionally, bamboo fibers possess relatively high tensile strength and flexibility, which help distribute bending stresses more evenly across the fibers through stretching and reduce the initiation and propagation of cracks [20].

Intermediate flexural strengths of 147 ± 7 and 162 ± 6 MPa are found in the laminates dominated by flex banner and fiberglass (V3 and V4, G/F/F/F/G), respectively. These values represent increases of about 100% (V3) and 121% (V4) compared to the neat epoxy. The addition of 1 wt% CaCO_3_ results in an approximately 10 percent increase between V3 and V4, indicating that the matrix is becoming denser and the fiber–matrix interface is strengthening. However, although fiberglass contributes significantly to stiffness, its relatively brittle nature limits the capacity of these laminates to accommodate large deformations under bending, so their strengths remain below that of the bamboo-rich V2 [54].

The mixed hybrid configurations combining bamboo, flex banner, and fiberglass (V5–V8) showed lower flexural strength overall. For the G/F/B/F/G sequence, V5 and V6 recorder 61 ± 3 MPa and 72 ± 3 MPa, respectively. With CaCO_3_ addition, flexural strength increased by approximately 19%, and V6 became comparable to neat epoxy. For the G/B/F/B/G sequence, V7 and V8 recorded 128 ± 7 and 111 ± 4 MPa, respectively. In this case, the introduction of CaCO_3_ was associated with a decrease of around 13%, suggesting that the filler may not be optimally dispersed or that it exacerbates stress concentrations at already heterogeneous interfaces. Overall, the flexural strength of these hybrid lay-ups remained up to 74% higher than that of neat epoxy (V7 vs. V9), but was lower than that of the bamboo-dominant and flex-dominant laminates.

The reduced flexural performance of the hybrid laminates can be attributed to interfacial incompatibilities between the natural bamboo fibers and the synthetic fiberglass/flex banner layers, which can cause asymmetric stress distribution and localized failure initiation under bending [55]. Differences in surface energy, morphology, and wettability hinder uniform resin wetting across the different reinforcements, leading to regions of weaker bonding.

The flexural response was influenced by laminate consolidation and effective fiber content. Compared with V7, V8 showed a higher void fraction and a lower fiber volume fraction (Figure 7), which may reduce load transfer efficiency during bending and leads to lower flexural strength. The impact response was governed by different mechanisms. CaCO_3_ addition may promote energy dissipation through crack deflection and increased interfacial friction, together with delamination and fiber pull-out. Therefore, V8 may exhibit higher impact strength even when its flexural strength was reduced.

### 3.5. Hardness Analysis

**Figure 10:**
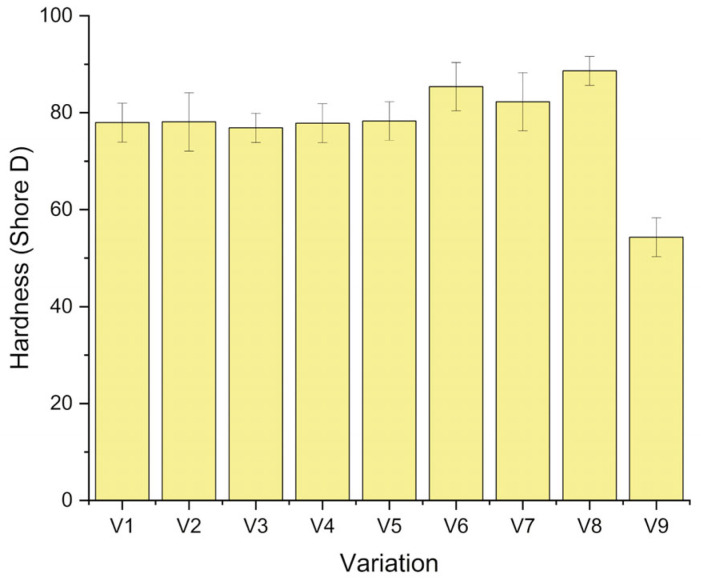
Hardness value of the composite.

Figure 10 shows the Shore D hardness of the laminates for the different stacking sequences and CaCO_3_ contents. The measured hardness values range from about 54 to 89 Shore D. The neat epoxy (V9) showed the lowest hardness, 54 ± 4 Shore D. In contrast, all reinforced composites showed higher values, suggesting that the presence of fibers and filler increased the surface resistance to indentation. Compared with neat epoxy, the hardness of the reinforced laminates increases by approximately 41–63%. This trend indicates that reinforcement improved surface rigidity under the present test conditions.

For the bamboo-dominant configuration G/B/B/B/G (V1, V2), the hardness values were 78.0 ± 4 (V1) and 78.1 ± 6 (V2), i.e., approximately 43–44% higher than that of neat epoxy. The minimal difference between V1 and V2 (≈0.2%) suggests that the addition of 1 wt% CaCO_3_ had little influence on hardness when bamboo dominates the core, and the outer surfaces are still glass fiber. Similar behavior was observed for the flex-banner/fiberglass-dominated sequence G/F/F/F/G (V3, V4), where the hardness was 76.9 ± 4 and 77.8 ± 3 Shore D, respectively. The increase due to 1 wt% CaCO_3_ was about 1.3%, indicating that the matrix in these laminates was already relatively dense and that the small filler addition mainly affects local microvoids rather than the global surface response.

A more pronounced effect of CaCO_3_ was observed in the mixed hybrid laminates. For the G/F/B/F/G sequence (V5, V6), hardness increases from 78.3 ± 5 to 85.4 ± 7 Shore D when 1 wt% CaCO_3_ is added, corresponding to an improvement of about 9%. For the G/B/F/B/G sequence (V7, V8), the hardness increases from 82.2 ± 5 to 88.6 ± 3 Shore D, representing a roughly 8% rise. In both cases, CaCO_3_-filled laminates (V6 and V8) exhibit substantially higher hardness than their unfilled counterparts and the neat epoxy, with overall gains of about 57% (V6) and 63% (V8) relative to V9. These results suggest that, in more complex hybrid lay-ups, the filler plays a more significant role in increasing local matrix stiffness and enhancing the integrity of the surface region by filling microvoids and strengthening the fiber–matrix interphase. Despite having identical fiberglass outer plies, the hardness variation among laminates may be governed by the subsurface fiber–matrix–void distribution and stacking sequence, which control the effective backing stiffness during indentation, as well as by the presence of CaCO_3_ filler that contributes to matrix stiffening and improved load support beneath the surface [56].

### 3.6. Morphological Analysis

**Figure 11:**
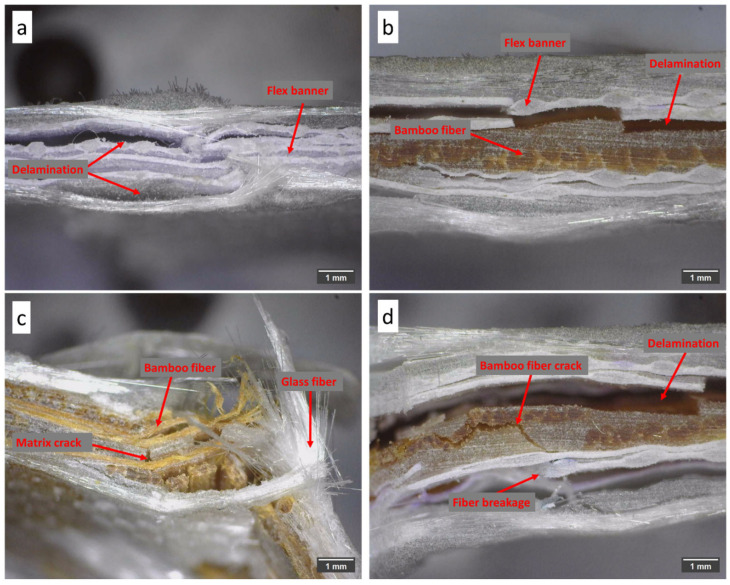
Fractographic observations after impact testing under the same applied impact energy: (**a**) delamination in V3, (**b**) interlayer separation between the flex banner and bamboo layers in V5, (**c**) matrix cracking in V2, and (**d**) fiber cracking in the bamboo layer in V6.

The morphology of the fracture surfaces of the hybrid composites reinforced with bamboo fiber, glass fiber, and flex banner after the impact test is shown in Figure 11. The observation was used to identify the most prevalent failure mechanisms that occurred as a result of the applied impact load. In Figure 11a, it can be observed that delamination occurred between the flex banner layers. This separation suggests weak interfacial bonding between layers, likely due to a lack of resin impregnation or a disparity in the surface energy ratio between the polymeric layers. Such delamination may contribute to impact energy dissipation through interlaminar separation rather than matrix plastic deformation [57].

A similar process of delamination between the flex banner and bamboo layers is shown in Figure 11b. This interfacial failure indicates that the resin wettability of bamboo may be limited, offering poor bonding strength. Delamination of these interfaces may promote crack propagation and reduce the overall impact resistance of the laminate. Figure 11c fracture morphology indicates that the matrix cracking and fiber pull-out occurred in the area of the glass fibers. The observed matrix cracks may be associated with stress concentrations generated by the stiffness difference between bamboo and glass fiber. The fiber pull-out indicates that a portion of the impact energy was absorbed due to the frictional sliding of the fibers, resulting in energy dissipation and toughness [11].

Meanwhile, Figure 11d shows bamboo layer fiber cracking, in addition to interlaminar delamination. The fracture mechanism appeared to propagate along the bamboo fibers, indicating that it exhibits a brittle failure behavior characteristic of natural fibers with a hollow microstructure [12]. Fiber cracking and delamination, when combined, imply that several intersecting failure modes occur during impact, resulting in a more complex energy absorption process.

**Figure 12:**
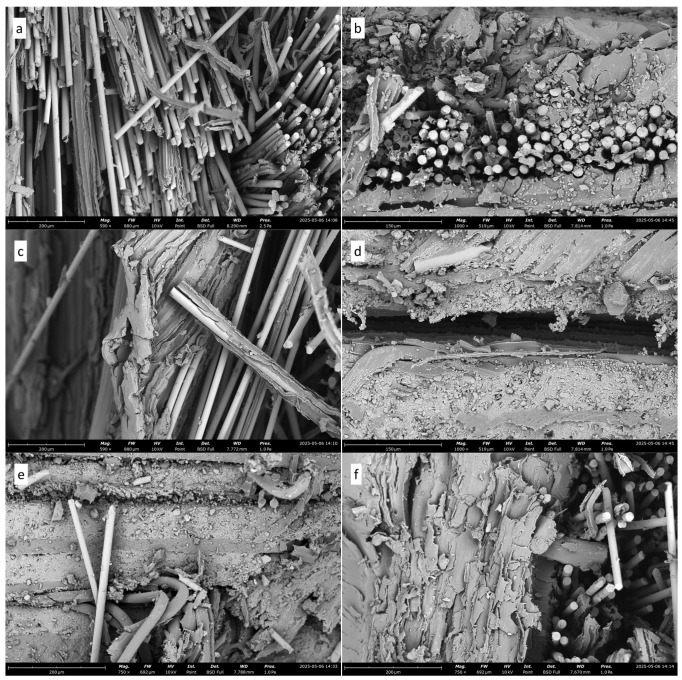
SEM micrographs of fractured surfaces of hybrid composites containing CaCO_3_: (**a**) glass fiber distribution in the matrix; (**b**) resin-free regions and voids near glass fibers; (**c**) glass fiber–bamboo fiber interface with good adhesion; (**d**) delamination in the bamboo fiber region; (**e**) fiber pull-out at the flex banner–glass fiber interface; and (**f**) partial debonding and cavities at the glass fiber–bamboo fiber interface.

Figure 12 presents SEM micrographs of the fractured surfaces of composites reinforced with glass fiber, bamboo fiber, and flex banner and containing CaCO_3_ filler. In Figure 12a, the distribution of glass fibers is visible as they are dispersed throughout the matrix, forming the primary reinforcement structure. But in Figure 12b, the existence of blank spaces and resin-free areas in the vicinity of the glass fibers proves that the process of resin impregnation during the manufacturing process was not uniform. These cracks can also serve as stress concentrators, which trigger crack propagation during mechanical loading [1].

Figure 12c displays the interface of the glass fiber–bamboo fiber. The matrix remains firmly bonded to the surface of the glass fiber after testing, indicating good interfacial adhesion and mechanical interlocking between the fiber and the resin. Figure 12d, however, shows that there is an evident delamination within the environment of the bamboo fiber area, meaning that the bonding of the bamboo fiber and the matrix is less intense than the glass fiber [26]. This delamination may occur due to the difference in properties between the surfaces of the fibers and their hydrophilicity, which affects the adhesion that develops during the curing process [20]. The interfacial behavior of different types of fiber is also shown in Figure 12e,f. The flex banner and glass fiber layers arrangement is observed in Figure 12e, Fiber pull-out was observed on the fracture surface, particularly in the glass fiber region. This effect is evidence of interfacial failure, which is caused by high shear stress during testing. Figure 12f illustrates the contact between the glass fiber and the bamboo fiber, which tends to debond partially, and exhibits bonding behavior in several different ways. Microstructural features such as cavities, delamination, and fiber pull-out were observed. These observations help explain the overall cracking behavior of the hybrid composite and indicate the influence of CaCO_3_ on interfacial bonding and material strength [55]. Table 4 summarizes a quantitative comparison of the flexural and impact properties of hybrid composites reported in the literature and those obtained in the present study.

## 4. Conclusions

This work indicates that both the laminate stacking sequence and the inclusion of CaCO_3_ filler impact the mechanical response of epoxy-based hybrid composites. Across all configurations tested, laminates with glass outer layers and a bamboo-rich core tended to exhibit better load sharing and damage tolerance. The G/B/B/B/G laminate with 1 wt.% CaCO_3_ (V2) had the greatest flexural and impact strengths in this study, whereas the G/F/B/F/G laminate without filler (V5) had the lowest values, perhaps due to less efficient interfacial bonding and stress transmission. Under the current test circumstances, all reinforced laminates outperformed clean epoxy (V9) in terms of flexural strength and impact. The addition of 1 wt.% CaCO_3_ increased interfacial cohesiveness and decreased crack-growth susceptibility. The G/B/B/B/G laminate with 1 wt.% CaCO_3_ demonstrated a positive strength–toughness balance, making it suitable for applications requiring both properties.

Future research should assess performance in genuine service scenarios. This should involve controlled aging at high temperatures, thermal cycling, and UV exposure in order to measure mechanical property retention and define deterioration processes. Moisture-related effects should be investigated using immersion and humidity conditioning, followed by diffusion analysis and mechanical testing to identify the effects on interfacial integrity, dimensional stability, and damage progression. Long-term behavior should also be assessed utilizing fatigue (flexural/tensile and, where applicable, impact fatigue), creep, and stress relaxation under various temperature–humidity combinations, as well as periodic measurements of residual flexural and impact characteristics.

## Figures and Tables

**Table 1:** Comparison of related studies and the present work.

Ref(s).	Research Focus	Key Focus	Limitation
[5,7]	Woven bamboo laminates	Bamboo reinforcement can improve load transfer and laminate performance.	Not compared directly with recycled flex banner + fiberglass in one framework.
[8]	Recycled flex banner interlayers	Flex banner interlayers enable waste utilization and can reinforce laminates.	Usually studied without bamboo–fiberglass hybrid stacking comparison.
[9,10]	Fiberglass in hybrid laminates	Fiberglass improves surface strength/stiffness and service stability.	Limited integration with recycled flex banner and bamboo in a unified laminate study.
[10,14,15,16,17,18]	Stacking-sequence effects	Mechanical response and failure depend strongly on layer arrangement.	Few studies evaluate stacking × mixed reinforcements together.
[19,20,21,22,23]	CaCO_3_-filled epoxy	Effects depend on dispersion/content and can influence interfacial behavior.	Limited laminate-level studies combining CaCO_3_ with stacking-sequence variation.

**Table 2:** Specimen variations in this study.

Sample Code	Stacking Sequence	CaCO_3_ (%)
V1	G/B/B/B/G	0
V2	G/B/B/B/G	1
V3	G/F/F/F/G	0
V4	G/F/F/F/G	1
V5	G/F/B/F/G	0
V6	G/F/B/F/G	1
V7	G/B/F/B/G	0
V8	G/B/F/B/G	1
V9	Epoxy	0

**Table 3:** Density change for identical stacking configurations with and without 1 wt.% CaCO_3_.

Stacking Group	Without CaCO_3_	With 1 wt.% CaCO_3_	Density Without (g/cm^3^)	Density with (g/cm^3^)	ΔDensity (g/cm^3^)	%Δ
G/B/B/B/G	V1	V2	1.1087	1.1499	0.0411	3.7
G/F/F/F/G	V3	V4	1.4894	1.5332	0.0437	2.9
G/F/B/F/G	V5	V6	1.4791	1.4815	0.0023	0.2
G/B/F/B/G	V7	V8	1.4069	1.4480	0.0411	2.9

**Table 4:** Quantitative comparison of flexural and impact properties of hybrid composites reported in the literature and the present study.

Ref(s).	Matrix	Reinforcement	Filler	Manufacturing Method	Flexural Strength (MPa)	Impact Strength
[58]	Unsaturated polyester	E-glass CSM450 + WR800 + CSM450	CaCO_3_	Hand lay-up + vacuum bagging	284.74	14.35 kJ/m^2^ (Charpy)
[59]	HDPE	-	CaCO_3_ + fumed silica	Melt compounding + compression molding	81	90 J/m^2^ (Izod)
[60]	HDPE	PKS particles	CaCO_3_	Melt-compounding (single-screw extruder) + hot press	-	20.16 J (Impact energy)
[22]	Unsaturated polyester	Pine fiber	CaCO_3_	Casting technique	166.51	133.99 kJ/m^2^
[61]	Recycled polypropylene	Pine wood flour	CaCO_3_	Injection molding	41.79	5.43 kJ/m^2^
This work	Epoxy	Glass/Bamboo/Flex banner	1 wt.% CaCO_3_	VARI	191	0.766 J/mm^2^

## Data Availability

The data can be made available upon request to the authors.

## References

[1] Nugraha A.D., Nuryanta M.I., Sean L., Budiman K., Kusni M., Muflikhun M.A. (2022). Recent Progress on Natural Fibers Mixed with CFRP and GFRP: Properties, Characteristics, and Failure Behaviour. Polymers.

[2] Hamdi L., Asma B., Ali B. (2024). Tensile mechanical performance of natural/natural fiber reinforced hybrid bio-composite materials—A statistical approach. J. Ind. Text..

[3] Hasan K.F., Al Hasan K.N., Ahmed T., György S.-T., Pervez N., Bejó L., Sándor B., Alpár T. (2023). Sustainable bamboo fiber reinforced polymeric composites for structural applications: A mini review of recent advances and future prospects. Case Stud. Chem. Environ. Eng..

[4] Hidalgo-Salazar M.A., Correa-Aguirre J.P., Román A.J., Gonzalez R., Vera R., Osswald T.A. (2025). Colombian natural fibers: Potential applications in sustainable natural fiber reinforced composite materials. Polym. Compos..

[5] Kelkar B., Shukla S., Nagraik P., Paul B. (2023). Structural bamboo composites: A review of processing, factors affecting properties and recent advances. Adv. Bamboo Sci..

[6] Nurhania N., Syarifuddin S., Armynah B., Tahir D. (2023). Fiber-reinforced polymer composite: Higher performance with renewable and eco-friendly plant-based fibers. Polym. Renew. Resour..

[7] Shi J., Wu Y., Zhang M., Zhang J., Zhang W., Chen H., Peng Y., Shi S.Q., Xia C. (2024). Bamboo fiber-reinforced epoxy composites fabricated by vacuum-assisted resin transfer molding (VARTM): Effect of molding sequence and fiber content. Polym. Compos..

[8] Widodo R.D., Nuryanta M.I., Gumelar M.H., Rohman S., Mujaki A., Darsono F.B., Muflikhun M.A. (2025). Impact and Flexural Response of Hybrid Composite Consisting of Waste Flex Banner and Glass Fiber. Jordan J. Mech. Ind. Eng..

[9] Saatcioglu K., Venkatraman P.D. (2024). The environmental impact of end-of-life PVC flex banners and its potential upcycling opportunities. Waste Manag. Bull..

[10] Uttaravalli A.N., Dinda S., Gidla B.R. (2021). Potential applications of Post-Consumer Vinyl Flex Banner (PCVFB) materials: Sustainable management approach. Int. J. Sustain. Eng..

[11] Nuryanta M.I., Aryaswara L.G., Korsmik R., Klimova-Korsmik O., Nugraha A.D., Darmanto S., Kusni M., Muflikhun M.A. (2023). The Interconnection of Carbon Active Addition on Mechanical Properties of Hybrid Agel/Glass Fiber-Reinforced Green Composite. Polymers.

[12] Nuryanta M.I., Sentanuhady J., Muflikhun M.A. (2022). Moisture absorption behavior of hybrid composite laminates consist of natural and glass fiber. Mater. Today Proc..

[13] Flaifel M.H., Shahdan D., Mhareb M.H.A., Ahmad S.H., Alghamdi A.A.A., Alajerami Y.S., Sayyed M.I. (2024). Unveiling enhanced properties of sustainable hybrid multifunctional graphene nanoplatelets incorporated polylactide/liquid natural rubber/polyaniline bio-nanocomposites for advanced radiation and particle shielding applications. J. Mater. Sci..

[14] Xu D., He S., Leng W., Chen Y., Wu Z. (2023). Replacing Plastic with Bamboo: A Review of the Properties and Green Applications of Bamboo-Fiber-Reinforced Polymer Composites. Polymers.

[15] Zhuo H., Dong X., Liu Q., Hong L., Zhang Z., Long S., Zhai W. (2025). Bamboo-inspired ultra-strong nanofiber-reinforced composite hydrogels. Nat. Commun..

[16] Saatcioglu K., Venkatraman P.D. (2025). Environmental impact, economic and carbon footprint assessment of end-of-life PVC flex banners and its potential upcycling opportunities in the fashion industry. Sci. Total Environ..

[17] Shaik M.S., Subramanian H.S., B. R.K., Suyambulingam I., Senthamaraikannan P., Kumar R. (2025). A Review on Fiber Properties, Manufacturing, and Crashworthiness of Natural Fiber-Reinforced Composite Structures. J. Nat. Fibers.

[18] Thapliyal D., Verma S., Sen P., Kumar R., Thakur A., Tiwari A.K., Singh D., Verros G.D., Arya R.K. (2023). Natural Fibers Composites: Origin, Importance, Consumption Pattern, and Challenges. J. Compos. Sci..

[19] Oladele I.O., Falana O.S., Okoro C.J., Onuh L.N., Akinbamiyorin I., Akinrinade S.O., Adegun M.H., Odemona E.T. (2025). Sustainable composites reinforced with glass fiber and bio-derived calcium carbonate in recycled polypropylene. Hybrid Adv..

[20] Flaifel M.H. (2020). An Approach Towards Optimization Appraisal of Thermal Conductivity of Magnetic Thermoplastic Elastomeric Nanocomposites Using Response Surface Methodology. Polymers.

[21] Afiefudin M., Widodo R.D., Rusiyanto R. (2023). Widodo, Fabrication and Characterization of Asbestos Free Brake Pads. Automot. Exp..

[22] Gapsari F., A. M.S., Putri T.M., Juliano H., Djakfar L., Handajani R.P., Budio S.P., Juwono P.T., Jagadeesh P., Rangappa S.M. (2022). Influence of calcium carbonate fillers on pine fiber reinforced polyester composites. Polym. Compos..

[23] Nuryanta M.I., Widodo R.D., Mujaki A., Rusiyanto, Kriswanto, Widayat W., Fitriyana D.F., Firmansyah H.N., Darsono F.B., Muflikhun M.A. (2024). The effect of stacking sequence on the properties of hybrid agel/glass fiber reinforced polymer composite laminates. IOP Conf. Ser. Earth Environ. Sci..

[24] Longkaew K., Gibaud A., Tessanan W., Daniel P., Phinyocheep P. (2023). Spherical CaCO_3_: Synthesis, Characterization, Surface Modification and Efficacy as a Reinforcing Filler in Natural Rubber Composites. Polymers.

[25] Nuryanta M.I., Nurhary M.A., Firmansyah H.N., Joshua D., Hajad M., Widodo R.D., Widodo T.D., Kusni M., Wiranata A., Kusumawanto A. (2025). Optimization of Kaolin Clay Composition for Enhanced Mechanical Properties in 3D-Printed Structures. Constr. Mater..

[26] Ming L., He H., Li X., Tian W., Zhu C. (2024). Study of the Effect of NaOH Treatment on the Properties of GF/VER Composites Using AE Technique. Materials.

[27] Thandavamoorthy R., Devarajan Y., Thanappan S. (2023). Analysis of the characterization of NaOH-treated natural cellulose fibre extracted from banyan aerial roots. Sci. Rep..

[28] Shi J., Zhang W., Zhang J., Yuan S., Chen H. (2023). Thermal Properties and Void Characteristics of Bamboo Fiber-Reinforced Epoxy Resin Composites Prepared by Vacuum-Assisted Resin Transfer Molding Process. J. Nat. Fibers.

[29] Tosto C., Saitta L., Barouni A., Sarasini F., Tirilló J., Bavasso I., Ziegmann G. (2024). Comparison of carbon-reinforced composites manufactured by vacuum assisted resin infusion with traditional and fully recyclable epoxy resins. Polym. Compos..

[30] Shi Y., Wang B., Du K., Liu Y., Kang R., Wang S., Zhang J., Gu Y., Li M. (2025). Process Monitoring for Vacuum-Assisted Resin Infusion by Using Carbon Nanotube-Based Sensors. Polymers.

[31] Wang T., Huang K., Guo L., Zheng T., Zeng F. (2023). An automated vacuum infusion process for manufacturing high-quality fiber-reinforced composites. Compos. Struct..

[32] (2025). Standard Test Methods for Determining the Izod Pendulum Impact Resistance of Plastics.

[33] (2017). Standard Test Methods for Flexural Properties of Unreinforced and Reinforced Plastics and Electrical Insulating Materials.

[34] (2021). Standard Test Method for Rubber Property—Durometer Hardness.

[35] Grisin B., Carosella S., Middendorf P. (2024). Vacuum Chamber Infusion for Fiber-Reinforced Composites. Polymers.

[36] Ismail A.S., Jawaid M., Hamid N.H., Yahaya R., Sain M., Sarmin S.N. (2022). Dimensional stability, density, void and mechanical properties of flax fabrics reinforced bio-phenolic/epoxy composites. J. Ind. Text..

[37] Praveena B.A., Santhosh N., Buradi A., Srikanth H.V., Shankar G., Ramesha K., Manjunath N., Karthik S.N., Naik M.R., Kumar S.P. (2022). Experimental Investigation on Density and Volume Fraction of Void, and Mechanical Characteristics of Areca Nut Leaf Sheath Fiber-Reinforced Polymer Composites. Int. J. Polym. Sci..

[38] Shen R., Liu T., Liu H., Zou X., Gong Y., Guo H. (2024). An Enhanced Vacuum-Assisted Resin Transfer Molding Process and Its Pressure Effect on Resin Infusion Behavior and Composite Material Performance. Polymers.

[39] Zuhudi N.Z.M., Zulkifli A.F., Ishak F.A., Aris K.D.M. (2023). Selecting the appropriate method for the void and moisture content measurement of fibre reinforced composites: A review. AIP Conf. Proc..

[40] Widodo R.D., Nuryanta M.I., Fitriyana D.F., Rusiyanto, Firmansyah H.N., Widayat W., Kriswanto, Darsono F.B., Mujaki A., Muflikhun M.A. (2025). The influence of flex banner types on the impact and flexural strength of hybrid composite materials. J. Phys. Conf. Ser..

[41] Ghosh I., Sharma C., Tandon R. (2020). Structural evaluation of chitosan-modified precipitated calcium carbonate composite fillers for papermaking applications. SN Appl. Sci..

[42] Lian X., Mou W., Kuang T., Liu X., Zhang S., Li F., Liu T., Peng X. (2020). Synergetic effect of nanoclay and nano-CaCO_3_ hybrid filler systems on the foaming properties and cellular structure of polystyrene nanocomposite foams using supercritical CO_2_. Cell. Polym..

[43] Thirupathi S., Gopalan V., Mallichetty E. (2025). Investigation of void content in Borassus flabellifer fiber/epoxy bio-nanocomposite using hyperparameter tuned ANN and response surface methodology optimisation. Sci. Rep..

[44] Magunga L., Mofokeng T.G., Motloung M.T., Ncube P., Mochane M.J. (2025). The Effect of Calcium Carbonate on the Flame Retardancy, Thermal Stability, and Dynamic Mechanical Properties of the PLA/Maize Stalk Composites. Fibers Polym..

[45] Dev B., Rahman A., Nipu S.A., Habiba S.U., Rahman S., Ahmed F., Rahman Z. (2025). Hybrid bamboo-banana-cotton sandwich composites for lightweight roofing: Mechanical, thermal and microstructural analyses. Hybrid Adv..

[46] Abhilash R., Venkatesh G., Chauhan S.S. (2021). Development of bamboo polymer composites with improved impact resistance. Polym. Polym. Compos..

[47] Wen X., Fu K., Dou Y., Xia X., Zhang J. (2022). Stiffness and Frequency Response Characteristics of Glass Fiber Reinforced Plastic Wave Springs with Different Periods and Its Finite Element Analysis. Materials.

[48] Asmare S., Yoseph B., Jamir T.M. (2023). Investigating the impact resistance of E-glass/Polyester composite materials in variable fiber-to-matrix weight ratio composition. Cogent Eng..

[49] Zhang Z., Zhan T., Ren J., Peng Y., Cao J. (2025). Green Functionalization of CaCO_3_ via Biobased Modifiers: A Sustainable Strategy to Enhance Interfacial and Mechanical Behaviors in Bamboo-Plastic Composites. Polym. Compos..

[50] Ismail A.S., Jawaid M., Sarmin S.N., Fouad H., Khiari R., Zainudin E.S. (2025). Physical, mechanical, and thermal properties of epoxy composites with woven kenaf and kenaf/cotton fabrics. Cellulose.

[51] Khafidh M., Putera F.P., Yotenka R., Fitriyana D.F., Widodo R.D., Ismail R., Irawan A.P., Cionita T., Siregar J.P., Ismail N.H. (2023). A Study on Characteristics of Brake Pad Composite Materials by Varying the Composition of Epoxy, Rice Husk, Al_2_O_3_ and Fe_2_O_3_. Automot. Exp..

[52] Dong C. (2024). Flexural properties and optimisation of hybrid composites reinforced by carbon, glass and flax fibres. Hybrid Adv..

[53] dos Santos A.J.G., Ribeiro M.M., Corrêa A.d.C., Rodrigues J.d.S., Silva D.S., Junio R.F.P., Monteiro S.N. (2025). Investigation of the Flexural and Tensile Properties of Hybrid Polyester Composites Reinforced with Bamboo Fibers and Red Mud Waste. Polymers.

[54] Ramachandran K., Khan M., Perera R.A.T., Jayaseelan D.D. (2025). Tensile and flexural behavior of synthetic and hybrid natural fiber composites for lightweight applications. Polym. Compos..

[55] Webb C., Qi K., Anguilano L., Rivera X.S. (2024). Mechanical and environmental evaluation of ground calcium carbonate (CaCO_3_) filled polypropylene composites as a sustainable alternative to virgin polypropylene. Results Mater..

[56] Wen Y., Wang Z., Yuan X., Yang X. (2025). Optimization of Mechanical Properties and Durability of Steel Fiber-Reinforced Concrete by Nano CaCO_3_ and Nano TiC to Improve Material Sustainability. Sustainability.

[57] Dasiewicz J., Kowaluk G. (2025). Upcycling Calcium Carbonate as an Alternative Filler in Layered Wood Composite Technology. Materials.

[58] Setyanto D., Jayatun Y.A., Basoeki P.D., De Fretes A. (2022). Physical Properties of Glass-Fibre-Reinforced Polymer Filled with Alumina Trihydrate and Calcium Carbonate. Polymers.

[59] Alshammari B.A., Alenad A.M., Al-Mubaddel F.S., Alharbi A.G., Al-Shehri A.S., Albalwi H.A., Alsuabie F.M., Fouad H., Mourad A.-H.I. (2022). Impact of Hybrid Fillers on the Properties of High Density. Polymers.

[60] Frimpong E.K., Kayaba A.-M., Akromah S., Nettey-Oppong E.E., Mensah E.E., Issakah O., Asare E. (2025). Development and characterization of sustainable PKS/CaCO_3_/HDPE hybrid composites for enhanced thermal and mechanical performance. Compos. Adv. Mater..

[61] Kavas E., Terzioğlu P. (2025). Calcium carbonate’s impact on pine wood flour and talc-filled recycled polypropylene composites for sustainable material applications. J. Compos. Mater..

